# Mechanisms of cancer cell death induction by triptolide: A comprehensive overview

**DOI:** 10.1016/j.heliyon.2024.e24335

**Published:** 2024-01-10

**Authors:** Ke Feng, Xiaojiang Li, Yuzhuo Bai, Dawei Zhang, Lin Tian

**Affiliations:** a Department of General Surgery, Affiliated Hospital of Changchun University of Traditional Chinese Medicine, Changchun, 130000, China; b Department of Breast and Thyroid Surgery Affiliated Hospital of Changchun University of Traditional Chinese Medicine, Changchun, 130000, China; c Department of General Surgery Baishan Hospital of Traditional Chinese Medicine, Baishan, 134300, China; d Department of Lung Oncology, Affiliated Hospital of Changchun University of Traditional Chinese Medicine, Changchun, 130000, China

**Keywords:** Triptolide, Cancer mechanisms, Apoptosis, Targeting, Anti-tumor

## Abstract

The need for naturally occurring constituents is driven by the rise in the cancer prevalence and the unpleasant side effects associated with chemotherapeutics. Triptolide, the primary active component of “Tripterygium Wilfordii”, has exploited for biological mechanisms and therapeutic potential against various tumors. Based on the recent pre-clinical investigations, triptolide is linked to the induction of death of cancerous cells by triggering cellular apoptosis via inhibiting heat shock protein expression (HSP70), and cyclin dependent kinase (CDKs) by up regulating expression of P21. MKP1, histone methyl transferases and RNA polymerases have all recently identified as potential targets of triptolide in cells. Autophagy, AKT signaling pathway and various pathways involving targeted proteins such as A-disintegrin & metalloprotease-10 (ADAM10), Polycystin-2 (PC-2), dCTP pyro-phosphatase 1 (DCTP1), peroxiredoxin-I (Prx-I), TAK1 binding protein (TAB1), kinase subunit (DNA–PKcs) and the xeroderma-pigmentosum B (XPB or ERCC3) have been exploited. Besides that, triptolide is responsible for enhancing the effectiveness of various chemotherapeutics. In addition, several triptolide moieties, including minnelide and LLDT8, have progressed in investigations on humans for the treatment of cancer. Targeted strategies, such as triptolide conjugation with ligands or triptolide loaded nano-carriers, are efficient techniques to confront toxicities associated with triptolide. We expect and anticipate that advances in near future, regarding combination therapies of triptolide, might be beneficial against cancerous cells.

## 1 Introduction

With more than 2.6 million new cases each year, cancer poses a serious risk to the general population and is second largest cause of death worldwide [1]. According to research, a large number of genes that code for proteins like, growth-factor receptors, tumor-suppressors, growth-factors, transcription-factors and anti-apoptotic proteins are dysfunctional in the majority of malignancies. These proteins are all potential targets for cancer treatment [2]. The need for naturally occurring constituents is driven by the rise in the cancer prevalence and the unpleasant side effects associated with chemotherapeutics. In contrast, the idea of using organic components to prevent cancer is becoming prevalent, particularly since conventional targeted therapies already had limited therapeutic success [3].

Countless phytochemicals in the plant kingdom were initially employed as herbal remedies in their raw forms as infusions, ointments and syrups. The World Health Organization (WHO) defines classic or traditional therapy as the use of herbal medicines in the primary healthcare resource and thus encompasses understanding, competence, and principles for the cure and avoidance of illnesses [4]. Traditional Chinese herbal remedies, Ayurvedic herbal remedies, Western herbal remedies, which originated in Rome and Greece and later expanded throughout Europe and North and South America, and traditional Arab medicines, which serves as the foundation for innovative and herbal therapies used nowadays, are the four primary systems of herbal remedies [5,6]. Nowadays, several effective plant-based compounds are present in about 25% across all prescription drugs [7].

Phytochemicals have exhibited considerable improvement in the treatment of a number of cancers. Plant-based secondary metabolites may provide an endless supply of molecules for the development of novel medications. Although the plant’s separated chemicals may not be used to make a medicine directly, it does lead to the creation of potentially innovative compounds [8]. The impacts of plant-based constituents on cell cycle regulation and apoptotic pathways have received a lot of attention recently, but not much is reputed concerning their influence on non-apoptotic pathways, such as mitotic-catastrophe, autophagy, senescence that leads to the death of cells and programmed-necrosis, also known as “necroptosis” [9].

Natural ingredients have been a crucial source for modern medications, particularly those originating from traditional medicines. The active components found in traditional medication have garnered considerable interest and have been a crucial source for certain medications that are frequently prescribed [10]. The “Tripterygium wilfordii Hook-F” (TwHF, usually regarded as Thunder God Vine), a vine species in the “Celastraceae” family that belongs to the genus “Tripterygium”, is primarily found in southern China, Korea, and Japan. TwHF has been used for two thousand years to cure human illness, including parasite infections, swellings, mounting-conglomerations, and breast abscesses. The establishment of numerous TwHF botanical extracts for clinical application was the result of centuries of research to optimize the effectiveness of TwHF. Clinical trials involving TwHF concentrates for the treatment of a number of ailments, such as ankylosing spondylitis, rheumatoid arthritis, kidney transplants, SLE (systemic lupus-erythematosus) along with number of skin infections, have been conducted [11].

About 400 botanical extracts, including celastrol, triptolide, wilfordine and wilforcidine, have been isolated and analyzed from TwHF in order to gain a more profound and considerate knowledge of TwHF intervention [12]. The most effective anti-proliferative compound, triptolide, was initially discovered in 1972 by Kupchan. It displayed nearly all of the medicinal potential of TwHF concentrates along with their significant toxicity. Triptolide’s biological mechanism and therapeutic potential have both been thoroughly studied from its very discovery. It was reported to have considerable positive therapeutic benefits, such as effects against malignancy, inflammation, rheumatoid arthritis, Alzheimer’s disease, osteoporosis, glaucoma, chronic asthma, anti-fertility, immunosuppression, and effects against cysts [11].

Tripterygium regelii, Tripterygium hypoglaucum, Tripterygium forrestii Loes and Tripterygium doianum are among the plant species in the genus “Tripterygium” that also contain triptolide in contrast to TwHF [11]. While being viewed as different species in herbal medicine, each of those species possess a comparable collection of natural bioactive compounds, and respective extracts have a similar range of pharmacological actions [13].

Interestingly, recent research has shown that triptolide is a potent anti-tumorigenic agent that could suppress the development of malignant cells within in-vivo and in-vitro settings, including glioblastoma, adrenal, breast, gastric and cancerous cells of pancreas [14,15]. Owing to triptolide’s potential to treat severe conditions like cancer, neurological disorders and immune disorders, investigators seems to be attempting to ascertain potential hazardous dosing range as well as creating new dosage forms in an effort to increase its therapeutic efficacy. We therefore have great expectations regarding triptolide as a promising new medication for the management of malignancy and CNS disorders in the future.

Meanwhile, significant obstacles still need to be addressed before triptolide may reach its full medical potential, including limited solubility in water, a constrained therapeutic window, multi organ toxic effects (such as urogenital, bone marrow, harming digestive, reproductive systems as well as blood circulatory) and toxicities based on innate mechanism. When use for long period of time, in certain complicated cases, triptolide causes significant side effects like liver damage, hepatomegaly and an increase in the serum levels of the aspartate aminotransferase (AST), superoxide dismutase (SOD) and alanine aminotransferase (ALT) [16]. Moreover, in recent years, the existence of cancer-stem-cells (CSCs) has been reported, owing to their ability to differentiate, production of identical cancer cells, higher risk of growth of cancerous cells and resistance to chemotherapeutic agents. Inhibition of various signaling pathways by triptolide, involved in CSCs propagation including Hedgehog (Hh), AKT and STAT pathway along with apoptosis, autophagy and development of strategies for targeted delivery of triptolide at CSCs site can be beneficial for eradicating cancerous cells [17]. Over the last few years, numerous research groups across the world have carried out an extensive cumulative synthesis [18] and structural modification of triptolide and its dependents, yielding a wealth of crucial SARs (structure activity relationships) data. The goal of these studies was to discover triptolide precursors with minimal toxicity and useful drug like attributes. Moreover, certain triptolide substitutes have made progress in human studies regarding the therapy of rheumatoid arthritis and malignancy. Examples are (5R)-5-Hydroxytriptolide (LLDT-8) and minnelide [19,20]. Targeted approaches, such as conjugating triptolide to ligands, which exclusively associate with cell-specific receptors, and encapsulating triptolide within nano-carriers, are other effective ways to diminish triptolide’s extreme toxic effects while preserving its effectiveness. Hence, relevant delivery techniques that had been created for the targeting of triptolide were examined in this review in order to get a thorough grasp of this field and offer recommendations for future research on triptolide. Based on this article, proposed research methodologies may be developed to advocate the broader use of triptolide or its variants in clinical settings, showcasing the importance of triptolide like a promising plant constituent that contributes to the development of safe and effective therapeutic interventions for a wider variety of medical conditions.

## 2 Pharmacological effects of triptolide against various tumors

### 2.1 Apoptosis-related anti-tumor effects

#### 2.1.1 HPS70

**Fig. 1:**
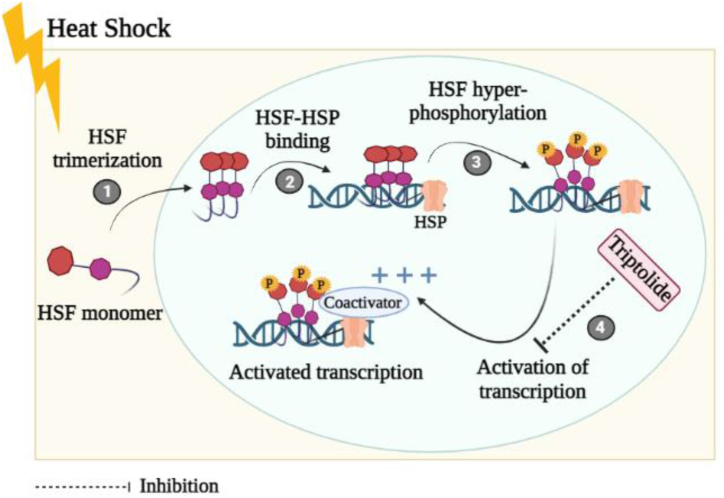
Heat shock response inhibition by triptolide: When exposed to heat shock, HSF1-monomer gradually develops transcriptional activity. In the first stage, HSF builds up in the nucleus & oligomerizes into a trimer that is capable of binding DNA. Trimeric HSF1 links to HSE sequences (HSF-HSP) found in the target genes' regulators in step 2. HSF1 is hyperphosphorylated in step 3. Step 4 involves the entire transcriptional activity of the hyperphosphorylated HSF1 and the activation of the heat shock gene. The heat shock response is inhibited by triptolide at the level of HSF1 transcriptional activation (Created with BioRender.com).

In many cancers, the HSP70s (70-kDa heat shock proteins), which aid in proper folding of protein and have a neuroprotective function, are up-regulated [14]. The importance of HSP70 inhibition in the treatment of diseases is illustrated by the fact that down-regulation of HSP70 employing siRNA or various indigenous or synthetic inhibitors can prevent, reduce, or indeed counteract malignant transformation [21]. According to recent research, triptolide’s anti-tumor efficacy is intimately linked with inhibition of HSP70 in a wide range of solid masses, particularly pancreatic malignancies [22]. According to statistical data, triptolide inhibits the HSP70 expression, improves activity of caspase-3, and triggers cytochrome-c release in both in-vitro and in-vivo settings without impacting the survival of healthy pancreatic cells [23]. Furthermore, triptolide suppresses the activity of HSP70 and inhibits glioblastoma growth without causing any adverse outcomes [24]. Moreover, it is yet unknown how triptolide works to inhibit HSP70. The inhibition of activity of HSP70 by triptolide is attributed to the significantly lower configuration of HSE & HSF1 composites. These held accountable for the transcriptional activity of HSP70 in cells (PANC-1/MiaPaCa-2) [25], despite the fact that triptolide (10 mM, 24 h) was proposed to impact transcription of HSP70 without trimerization of HSF1 and adhering022 to (heat shock element) HSE in cell cultures of human [26]. One such mechanism of inhibition of transcription by triptolide is demonstrated in Fig. 1. The control of transcriptional activity is significantly influenced by miRNAs. Researchers found that triptolide elevated the production of miRNA that is linked to HSP70 to suppress HSP70 in pancreatic cancerous cells, irrespective of heat shock factor-1 (HSF1) [27]. It is uncertain if triptolide suppresses HSP70 through HSF1 pathway consequently. Triptolide (100 nM, 24 h) also decreased Sp1's glycosylation, a key transcription that malignant cell overexpress due to protein modification. This in turn decreased the production of HSP70 and NF-κB, which eventually contributed to the death of the cancerous cells (S2-VP10 and S2-013) [28]. Furthermore, for the effective treatment of pancreatic cancer, intraperitoneal administration of triptolide has been listed at preclinical stage for inhibition of HSP70 [29]. The resistance developed by HSP90 and HSP70 blockers in radiotherapy overcame by combining these blockers with triptolide as reported in previous clinical studies [30]. Moreover, triptolide when given in high doses to inhibit HSP70, could potentially produce toxic effects in liver and kidneys, which still needed to be addressed in the future research studies. As, HSP70 is notorious for developing resistance to chemotherapy, the clinical studies suggested the combination therapy of triptolide either with gemcitabine or pemetrexed. However, owing to limited solubility profile of triptolide, now minnelide is in under consideration (phase 1) for safe combination therapy in HSP70 induced chemoresistance effects [31].

#### 2.1.2 Cell cycle and cancer

The cell cycle and apoptosis are both crucial for tumor cell progression. Triptolide directly inhibits all cyclins, with the exception of E-type cyclins, causing an interruption of cell cycle and reduced expression of COX-2 and VEGF in cells with colon cancer [32]. Moreover, P53 was linked to triptolide’s anti-cancer effects, and restoring P53 activation caused tumor cells to die [33]. Triptolide (10 nM, 3 h) promoted apoptosis in drosophila by activating JNK even in cancerous cells that are lacking P53 [34]. Further research verified that triptolide (5–150 nM for 24 h) enhanced p21 expression and concomitant association with CDKs to trigger halt in the phase G1, along with concurrent decline in phosphorylation of AKT and ERK in cancerous cells of the colon and rectum [35]. Triptolide (50–150 nM for 48 h) therapy is reported to decrease cell growth and impeded the G0/G1 transition in multiple-myeloma cells of human in a dose-dependent way via altering the demethylation of histone (JMJD2B and LSD1) [36]. Triptolide (120 nM for 24 h) was proposed to inhibit tumor growth by up-regulating P21wap or cip1, CDKs and P27-kip1, which are deleterious cell cycle regulators [37]. Triptolide (10–30 nM, 48 h) triggered halt in the G2 or M and S phases in contrast to the G0 or G1 phase in A375 cells (human melanoma) [38] via controlling NF-κB expression via Bcl-2 dysregulation and suppressing methyl-transferases and methylation of histone [39]. Additionally, inhibitory effects of triptolide at XPB ATPase specific targeted site on CDK12 and CDK13 (TFIIH subunit XPB) is under phase-I clinical trial in combination with paclitaxel for advanced stage solid tumor and HIV; and for effective treatment of pancreatic cancer, triptolide is under phase phase-II clinical trial [40].

##### 2.1.2.1 Pathways for AKT signaling

An important cell signaling system upstream of numerous genes that regulate metabolism, angiogenesis, and cell survival is the AKT/protein-kinase B signaling pathway [41]. Current research suggests that triptolide is responsible for a unique connection among AKT and p38, and trials with a p38 blocker confirmed this theory. In the context of a p38 blocker, triptolide’s capacity to stimulate p38, cause the degeneration of tRXR-α (a nuclear receptor for modulation of gene expression) and reverted TNF-α and suppress AKT [42]. Anti-apoptotic (Bik/Nbk, Bax and Bid) and Pro-apoptotic (Bid, Bax and Nbk/Bik) proteins make up Bcl-2 group, which is connected to cascade of AKT (Bcl-XL, Bcl-2, and Bcl-W) [38,43]. Triptolide suppresses Bcl2 to assert its anti-tumor action, according to prior studies. Furthermore, triptolide controlled the activity of Mcl1 (myeloid cell leukemin 1), a component of Bcl2 group included in preservation of the matrix of mitochondria and the suppression of release of the cytochrome-C, via triggering apoptotic cell death through a wide range of mechanisms (PI3K-AKT, MAPK, and JAK/STAT). It has also been observed that triptolide inhibits JAK2 transcription by causing the fragmentation of Mcl1 by caspase-3 [44], diminishing Bcl2 and Mcl1 levels in cells with chronic myeloid leukemia [45]. It’s interesting to note that triptolide (12.5–200 nM, 24–72 h) inhibited AKT and PI3K while also controlling HER2, which is abundantly expressed in many malignancies [46]. Triptolide’s ability to inhibit HER has not established, though. However, various triptolide’s anti-tumor targets have been summarized in Table 1.

**Table 1:** Triptolide’s targets for anti-tumor effects.

Targeting gene	Tumor cells	References
Caspases (3, 8 & 9)	Multiple-myeloma cells	[66]
XIAP	Acute-myeloid leukemia, cell lines for leukemia	[43]
bcr-abl	K562-cells	[67]
Bax, Bcl-2	HL-60, glioma cells,	[68]
p53, bax, p21 (cip1/waf1)	Gastric cancerous cells	[33]
NF-κB	Anaplastic thyroid cancer in humans	[69]
JNK-1/2, p38 MAPK, ERK-1/2, MKP1	NSCLC, hippocampal-cells	[70,71]
PI3K	Human fibro-sarcoma	[72]
HSP70	Pancreatic cancerous cells	[23]
5-LOX	Pancreatic cancerous cells	[59]
ADAM10	Cell lines for leukemia	[73]
RNA-polymerase	Cell line for human non-small cell lung-cancer	[74]
Mcl1, Jak2	Cells from human myelo-proliferative disorders	[44]
Histone methyl transferase	Myeloma	[75]
HER2	Cancerous cells of ovary	[46]
SENP1	PC-3 (cell lines)	[61]

#### 2.1.3 XIAP and MDM2

P53 dependent and independent methods are used by MDM2 as it is involved in development of malignancies. MDM2 associates with particular molecules to perform its anti-apoptotic activity. It is comprised of multiple mechanisms for controlling the integrity and suppressing the functioning of P53 [47]. Inhibiting expression, the IAP member of the group X linked inhibitor of apoptosis-protein (XIAP) operates as an anti-apoptotic agent [48]. It’s noteworthy that studies have shown that MDM2 controls the enhanced XIAP expression in malignancies, offering a fresh oncotherapy strategy [49]. Triptolide (25 nM for 24 h) pretreatment of U937 cells led to caspase-3 up-regulation and apoptosis, which were thought to be connected to XIAP suppression. Acute lymphoblastic-leukemia (ALL) cells showed comparable triptolide mediated XIAP suppression (10–100 nM, 24–48 h), which was correlated with a reduction in release of cytochrome *c* mediated by caspase-9 and Mcl1 via the mitochondrial apoptotic mechanism [43]. Furthermore, triptolide (25–100 nM, 24–48 h) and TRAIL (1–10 ng/mL, 24–48 h), a TNF having mild cytotoxic effects, were used to modulate XIAP suppression and increase killing of cancerous regions [50]. Additional study showed that triptolide (10–100 nM for 24 h) suppressed the MDM2 production and significantly suppressed XIAP via P53 independent way, restricting proliferation in ALL (acute-lymphocytic leukemia) cells [51]. These experiments demonstrated a decreased NF-κB expression via triptolide (a XIAP regulator). As a result, it’s apparent that triptolide controls MDM2 and/or NF-κB to modulate XIAP.

#### 2.1.4 RNA-polymerase II

Every eukaryotic cells include RNA-polymerase II (RPII), a polymerase that helps with transcription of gene as well as the transcription and development of mRNA. Triptolide blocked the entire genome, according to a prior study, without changing the adherence of transcription factors and DNA. Triptolide was discovered to decrease the gene transcription of RNA-polymerase II and the decomposition of short lived mRNA and RPB1 (a major component of RPII) [52]. Besides that, triptolide (200 nM, 1 h) phosphorylates RPB1 before it degrades, and this mechanism depends on the proteasome. Triptolide suppressed transcription of global-gene in the HeLa cells, and Rbp1 interacts with XPB to facilitate easy transcription [53]. Several parameters, like the phosphorylated carboxyl-terminal-domain (CTD), that modulates function of RPB1, control the ubiquitination of RPB1. Triptolide has previously been shown to regulate RPII breakdown by activating CDK7, which leads to the phosphorylation of Ser-5 [54].

#### 2.1.5 Additional systems

One of the recent study has shown the effectiveness of triptolide in deactivating hedgehog signaling pathway (Hh) that is involved in augmentation, apoptosis, expansion, relocation and viability of cancerous cells. Therefore, triptolide has proposed to be a novel deactivator of GLI against cancer of pancreas [55]. Furthermore, the suppression of pro angiogenic proteins (VEGFR-2 and Tie2) in relation to cancer progression was achieved following 100 nM triptolide administration to endothelial cells of human umbilical vein [56]. Moreover, in breast cancer cells (MCF-7), triptolide (1–40 ng/mL, 24–48 h) impacted the fragmentation of FAK (focal-adhesion-kinase) that is essential for adherence of cell and viability as well as depends on activation of caspase [57]. Subsequent research showed a reduced expression of MMP and raised E-cadherin via triptolide, which prevented the proliferation of cancer cells of ovary [58]. Arachidonic acid is metabolized when 5-lipoxygenase (5-LOX) is expressed exclusively in cancerous cells of pancreas. Inhibition of 5-COX by triptolide therapy led to anti-tumor action [59]. Triptolide (5 mg/kg) inhibited phosphorylation of theJNK (c-Jun-N terminal kinase), p38 & ERK in hippocampus, regulating the inflammatory response-related MAPK-phosphatase 1 (MKP1) enzyme [60]. Similarly, triptolide demonstrate excellent apoptotic action by suppressing SUMO-specific protease-1 expression in PC-3 cells (upon 7 days of treatment with triptolide; 5–30 nM) [61] as well as HIF1 production & levels of mRNA being inhibited in SKOV 3 cell (upon treatment with triptolide; 50–1000 nM for 12 h) [62]. Nevertheless, triptolide has been reported to create imbalance between T regulatory (Treg) and T helper 17 (Th17) cells, which are involved in regulating homeostasis modulated by innate immunity [63]. Such imbalance results in induction of autoimmune diseases along with the production of certain cytokines, thus contributing to hepatotoxicity [64]. However, by combination therapy of triptolide with quercitin, its toxic effects on liver can be reversed by regulating Treg/Th17 balance. Later, studies conducted on quercitin has revealed its impact on hepatitis induced by triptolide via Treg/Th17 balance modulation through TIM-3 pathway [65].

Furthermore, hyperthermia is gaining interest as an effective therapy for CSCs. Hyperthermia has been proposed to increase the temperature of cancerous cells to 42–45 °C, thereby subjected to induction of apoptosis (P53 and FAS genes), deactivating the cell cycle and activating immune system [17].

### 2.2 Route for anti-tumor signaling

#### 2.2.1 Processes of molecular signaling mediated by various proteins

Previously, it was hard to determine the targets of naturally active biological products, and nearly every approach of target recognition currently in use, including chromatographic techniques, comes with distinct drawbacks [76]. Throughout many years, a significant number of groups have worked to understand the molecular targets of triptolide. Triptolide has so far had shown to engage directly with 7 prime target-proteins, such as A-disintegrin & metalloprotease-10 (ADAM10), Polycystin-2 (PC-2), dCTP pyro-phosphatase 1 (DCTP1), peroxiredoxin-I (Prx-I), TAK1 binding protein (TAB1), kinase subunit (DNA–PKcs) and the xeroderma-pigmentosum B (XPB or ERCC3) [77]. We will examine each of the potential targets and associated biological significance in the subsequent sections.

##### 2.2.1.1 Polycystin-2 (PC-2)

Triptolide was shown to be localized on cell membranes, and Crews' group revealed in 2005 that triptolide’s affinity for putative targets increased at decreased calcium concentrations [78]. PC-2 is a (110-kDa) 3H-triptolide binding protein, whose genetic variation may cause ADPKD (autosomal-dominant polycystic kidney disease). In a kidney specific animal model, it was additionally shown that triptolide stimulated cellular Ca^2+^ eject across a PC-2 dependent route, inhibiting the tumor growth of kidney cancerous cells as well as reducing the mass of cyst [79]. Nevertheless, as it is unknown whether this calcium channel membrane controls overall tumor growth, it is unable to account for the strong tumor genesis effects of triptolide.

##### 2.2.1.2 A-disintegrin & metalloprotease-10 (ADAM10)

Marignani’s team discovered A-disintegrin & metalloprotease-10 (ADAM10) as a binding protein of triptolide using affinity chromatography and mass spectrometry [80]. An essential function of the ADAM10 (multi-domain transmembrane glycoprotein) in revascularization, fertilization, immunotherapy, and malignancy is to fragment and liberate the codomains of growth factors, cytokines and receptors [81]. Triptolide may limit ADAM10 overexpression and function together with ADAM10 knockdown to increase its activity. Unfortunately, there are no pharmacological studies that show how triptolide might impact ADAM10's enzymatic activity [73]. Triptolide is well recognized as having the capacity to hinder transcription. As a result, the suppression of modulation of ADAM10 could be linked to triptolide’s capacity to suppress general transcription, making ADAM10 a supplementary target of the compound. Triptolide also blocks the expression of ADAM10 at the level of transcription.

##### 2.2.1.3 dCTP pyro-phosphatase 1 (DCTPP1)

The triptolide binding protein dCTP pyro-phosphatase 1 (DCTPP1) was discovered in 2011 by Crews' team using a biotinylated photo cross-linked precursor of triptolide as just a probe [82]. DCTPP1 has been described as a “housekeeper protein” that controls the inter-cellular nucleotide array and blocks unauthorized nucleotides from integrating into DNA. The up regulation of DCTPP1 has been linked to a bad prognosis in a number of malignancies. However, DCTPP1 was disqualified as being a triptolide’s biological candidate for transcriptional activity, also for the suppression of propagation in both DCTPP1 up regulation and siRNA-mediated depletion studies [82]. However, multiple research teams showed how DCTPP1 antagonists had significant synergism with cytidine nucleotides (in cell line; HL60), via interrupting with nucleotide metabolism [83]. These DCTPP1 antagonists have an entirely different chemical structure than triptolide. It ought to be fascinating to construct DCTPP1 selective antagonists based on chemical architecture of the triptolide irrespective of effect of inhibition towards TFIIH/XPB. It also is significant; DCTPP1 is not a biologically appropriate target for triptolide’s anti-tumor effect due to its affinity for the protein being in the range of 100 mM, a factor far larger than its intracellular efficacy.

##### 2.2.1.4 TAK1 binding protein (TAB1)

In 2014, Shen’s team discovered that triptolide binds to TAK1 binding protein (TAB1) employing monoclonal antibody [84]. Inflammatory and immunological signaling pathways are thought to contain Transformational Growth-Factor B-Activated-Kinase 1 (TAK1), whose kinase efficiency is regulated by the protein TAB1 [85]. A disruption of this formation of complexes will hinder the subsequent signal transduction. When triptolide binds to TAB1 and it prevents the TAK1 and TAB1 complexes from forming, which reduces TAK1 expression. Un-phosphorylated MKK3/6 and MKK4 stop being able to phosphorylate p38 and JNK1/2, respectively, and the disinhibited TAK1 is unable to phosphorylate its compounds, MKK3/6 and MKK4, which inhibits the transcriptional activity and release of a variety of inflammatory cytokines involved in inflammatory and immune responses [84]. While the impact of triptolide on NF-kB and AP1 appears to be explained by the suppression of the TAK1- TAB1 linkage, this activity is unable to explain the anti-tumor effect of triptolide.

##### 2.2.1.5 Peroxiredoxin-I (Prx-I)

Crews' team employed a C-14 hydroxyl derived triptolide probe [82]; Yang’s team formed a C-13 derived triptolide probe and used it to identify peroxiredoxin-I (Prx-I), another triptolide binding protein [86]. Prx-I serves as a dual-purpose enzyme that catalyzes the hydrolysis of H_2_O_2_ in homo-dimers and behave like a molecular pacemaker in homo-decamers to avoid the degradation of its substrate proteins [87]. Prx-I’s Cys173 and Cys83 are covalently modified by triptolide, causing Prx-I decamers to dissociate. This inhibits Prx-I’s ability to function as a pacemaker without impacting its ability to function as a per-oxidase [86]. Adenanthin was discovered to have an intriguing complimentary impact on Prx-I; it just covalently changes Cys173 in Prx-I, specifically reducing the per-oxidase function of Prx-I without having a major impact on its pace making action [37]. Likewise as DCTPP1, Prx-I can’t entirely explain triptolide’s anti-cancer properties.

##### 2.2.1.6 Xeroderma-pigmentosum B (XPB or ERCC3)

A great deal of research has been done to understand how triptolide affects various cellular functions and signaling cascades [12]. Triptolide exhibits strong reduction of cell viability or propagation at the molecular level. Including the NCI60 array, it has been identified to be consistently efficient against several cell lines of cancer [74]. Triptolide appears to be a universal transcriptional inhibitor as evidenced by the up to 98% of regulated genes that were down regulated by relatively brief triptolide administration in human pan-genomic-DNA microarray tests [74]. Consequently, the suppressing effects of triptolide upon transcription was further confirmed via down regulation of wide range of proteins. These proteins are essential for apoptosis (Bcl-xL, Bcl-2, c-IAP1, Mcl-1, c-IAP2, XIAP), the cell cycle (cdc-25, cyclins D1, B1 and A1), anti-tumor (ADAM10, HSP70, p21, Myc, RNA polymerase, p27) and inflammatory activities and immuno-regulation (TNF-a, IL-1, IL-4, IFN-a, IL-6, IL-2, CD80, CD40) [88]. The binding domain for triptolide has been determined by using top down method to be xeroderma-pigmentosum B (XPB or ERCC3), a key component of the generic transcription factor (TFIIH). Researchers discovered that triptolide suppresses TFIIH’s DNA-dependent-ATPase activity, which in turn prevents RNA polymerase-II from mediating base excision fixing and activating transcription [88]. Several pieces of evidence pointed to the biological significance of XPB as the triptolide targeting molecule. Furthermore, a variety of triptolide variants with a broad range of therapeutic potential showed a significant association among the suppression of ATPase activity that depends on DNA and the restriction of propagation of the cell [88]. Also, discovery of the TFIIH/XPB as triptolide’s target molecule provides a mechanistic explanation for the majority of the drug’s negative consequences. The covalent alteration of XPB by triptolide, which takes place effectively within about an hour after triptolide exposure to cells, is not the only way that triptolide modulates generalized transcription. Triptolide causes conformational change and proteasome mediated depletion of the RNAP-II catalytic RPB1 component after a protracted incubation, which worsens its effects on RNAP-II signal transduction [52]. It has been debatable for a while whether triptolide and XPB’s covalent bonding is necessary for degradation of RPB1. Miao’s team discovered that decomposition and cellular growth impairment of RPB1 induced by triptolide was not alleviated by XPB down regulation by transient expression (siRNA) or by distinct XPB genetic variations [89]. They came to the conclusion that either triptolide initiates degradation of RPB1 or killing of cells do not depend on XPB [90]. Triptolide causes RPB1 to degrade, however the precise chemical mechanism behind this process is yet unclear.

##### 2.2.1.7 The DNA–PK complex as kinase subunit (DNA–PKcs)

The suppression of TFIIH/XPB, which is crucial for repair of nucleotide extirpation and DNA repair transcriptional regulation, is one cause of DNA damage induced by triptolide, involving nucleotides genetic mutations, breakage of double strand of DNA, & epigenetic changes [88]. By directly interacting with DNA-PKcs (kinase component of the DNA-PK complex), triptolide, according to Wan’s group, may alter integrity of the genome [91]. Triptolide inhibits DNA-PKcs' auto-phosphorylation on the Ser2056-residue after it binds to it, and un-phosphorylated DNA-PKcs is arrayed with hetero-dimer KU proteins at the extreme edge of DNA breakage. XPB is probably the most pertinent target among the several triptolide binding proteins that have been described so far, which explains both its anti-tumor efficacy and its potential toxicity. Several of the potential triptolide binding proteins that are still present are probably the result of off the mark target or side effects from TFIIH/XPB suppression. Although triptolide has a relatively poor affinity for most other targets, its backbone can be used as an initial point to improve its specificity and efficacy for some targets. Triptolide’s C12–13 epoxide unit is necessary for the suppression of XPB’s ATPase function [77]. To specifically target PC-2, DCTPP1, ADAM10, DNA-PKcs, and TAB1, triptolide conjugates without the C12–13 epoxide unit can be designed and screened.

### 2.3 Effects of triptolide on tumors via autophagy pathways

Autophagy is distinguished from apoptosis by accelerated, caspase independent lysosomal diminishment, organelle enlargement, karyopyknosis, and self-digestion [92]. Moreover, apoptosis and autophagy modulate one another via modest interaction, and a number of illnesses have connections between their signaling pathways [93]. Current research looked into whether autophagy and apoptosis are possible mechanisms by which triptolide destroys pancreatic cancerous cells. Triptolide (25–200 nM, 24–48 h) triggered two distinct mechanisms of cancerous pancreatic cells killing in various cell-lines; it is a potent drug effective against various tumors, as shown by the association of its activity with activation of autophagy (that depends on caspase) in S2-VP10 and S2-013 cells and apoptosis in Capan 1, Bx-PC3, and MiaPaCa 2 cells [94].

**Fig. 2:**
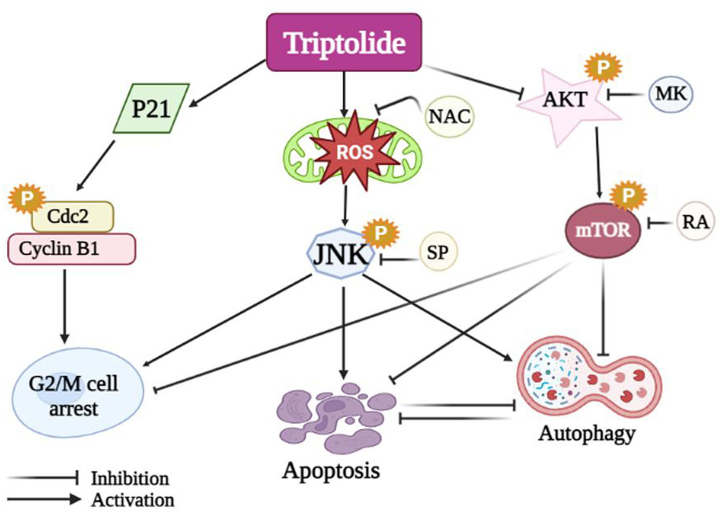
Apoptosis and autophagy induced by triptolide in glioma cells: a potential mechanism.

A number of molecular pathways mediates triptolide-regulated autophagy, with oxidative stress playing a significant role. In fact, triptolide can changed the regulation of a variety of genes implicated in the oxidative stress cascade, as shown by a genetic code microarray investigation [95]. Triptolide has inhibited the function of antioxidant enzymes, inappropriately elevated intrinsic reactive oxygen species levels (ROS), damaged mitochondria, elevated oxidative stress, and ultimately injured heart tissues or cardiomyocytes [96]. Oxidative stress can cause autophagy to start in order to destroy dysfunctional organelles and toxic proteins in cells [97]. Compromised mitochondria, known to be a major contributor of ROS in cardiomyocytes treated with triptolide, were specifically secluded for breakdown in acidic lysosomes [98]. As a result, it is believed that oxidative stress triggers triptolide-regulated autophagy, which clears the compromised mitochondria. Moreover, triptolide induced dysfunctional mitochondria in cardiomyocytes was prevented by rapamycin induced autophagy, which also lessened oxidative stress. Besides that, triptolide enhanced leukemia cell death via promoting the formation of ROS, which in turn induced autophagy [99]. Moreover, triptolide regulated autophagy in leukemia cells and other cell lines is significantly influenced by cytoplasmic-calcium, a major activator of autophagy [100]. In differentiating pheochromocytoma cell line, triptolide induced increased ROS levels and intracellular calcium produced by Aβ_25-35_, hence weakening autophagy. In contrast, triptolide triggered autophagy in glioblastoma cell line by raising ROS levels (Fig. 2) and intracellular levels of calcium [101]. Stimulation of autophagy by triptolide in cancerous cells of the pancreas was linked to an increase in intracellular levels of calcium. That mechanism was also regulated by persistent ER stress, suppression of the p70S6K/Akt/mTOR cascade, and activation of the signaling pathway of ERK [102]. Triptolide induced ER stress in prostate tumor cells, which then resulted in the release of free calcium into the cytoplasm. Subsequent activation of the β-(CaMKKβ)-AMPK signaling cascade by calcium led to the suppression of mTOR and the modulation of the PI3KIII and ULK1 subunits, which in turn led to the initiation of autophagy [103]. Within podocytes, triptolide increased autophagy and activated the mTOR-ULK1 pathway while down regulating the levels of *p*-mTOR, *p*-Akt, and *p*-ULK1 [104]. In triptolide regulated autophagy, additional processes have been investigated. The activation of ERK protein may be associated with stimulation of autophagy by triptolide in cancerous cells of the lungs [105]. Triptolide increased the p53 nuclear localization in Hela-cells, which then increased the production of the lysosomal-protein DNA-damage-regulated autophagy modulator (DRAM) and decreased the activity of mTORs. In the rat brain, triptolide improved autophagosome functioning by reducing the elevated mTOR levels of expression driven by ischemic injury [106]. Moreover, triptolide may have a particular mechanism of action in cardiomyocytes and cancerous cells of the breast in the lysosome [107]. Triptolide hence likely regulates autophagy by focusing on various machinery or signal routes.

### 2.4 Effects of synergism between triptolide and other cytotoxic drugs

#### 2.4.1 Triptolide promotes apoptosis induced by TNF family

Members of the NF-family cause apoptosis via p53 unrelated pathways. TNF’s activation of NF-κB, which results in pro inflammatory reactions, limits the cytotoxicity it causes in cells. In tumor cells, such as NCI–H1299 (null-p53) and A549 (wt-p53) cancerous cells of lungs [108] and cell lines of human cholangio-carcinoma, triptolide may inhibit TNF-α induced NF-κB signaling, enhancing the death of tumor cells triggered by TNF-α and limiting pro inflammatory reactions [109]. Moreover, triptolide greatly increases ERK2 stimulation and inhibits NF-κB expression to make cancerous cells of lungs more susceptible to TRAIL/Apo2L induced apoptosis [110]. Besides that, triptolide increases sensitivity to apoptosis induced by TRAIL in AML-cells via reducing p53 regulated DR5 and XIAP expression [111]. Triptolide may increase apoptotic cell death induced by TRAIL vulnerability in these TRAIL resistant cholangio-carcinoma cells of human [112]. An efficient treatment that causes cell death by apoptosis in pancreatic cancerous cells by activating caspase-9 and caspase-3 is complementary treatment with TRAIL and triptolide [113].

#### 2.4.2 Triptolide promotes apoptosis induced by chemotherapeutic agents

5-fluorouracil (5-FU) and triptolide both had growth-inhibiting effects on colon cancer and KB cancerous cells, but their cumulative effects were far more effective than their separate effects. While 5-FU and triptolide together showed synergistic action at lower dosages and encouraged apoptosis, they did not worsen chemotherapy’s adverse aspects [114]. Through stimulating expression of Bax and caspase-3 and suppressing expression of Bcl-2, triptolide instantaneously produces ROS, decreases NF-κB functioning, and makes cell lines of colorectal cancer susceptible to 5-FU [44]. In both KB-tax and KB-7D cells, triptolide suppresses the expression of MDR and multi-drug resistant protein. By triggering caspase-3 and inhibiting XIAP and Mcl1, it also causes apoptosis in these multi-drug resistant cancerous cells [44]. Combining triptolide with ionizing radiation (IR) for pancreatic tumors, cell viability decreased to 21% and tumor cell death increased as compared to individual treatment. Malignancy development in xenografts of AsPC-1in human cancerous pancreatic cells were further attenuated in the combined therapy of triptolide with IR when compared with just one therapy in-vivo. In both in-vitro and in-vivo models of pancreatic cancerous cells, triptolide and IR demonstrated synergistic anticancer potential [115]. Also, when nude mice were injected with human ovarian cancerous cells, triptolide improved the cytotoxic effects that were produced in culture by carboplatin and hence improved the carboplatin regulated suppression of cancer progression [116]. In cell lines of multiple-myeloma, triptolide was capable of augmenting the effects of PS-341/bortezomib or dexamethasone. In dexamethasone resistant (MM-1R) and dexamethasone sensitive (MM-1S) cells, triptolide promotes apoptosis. It’s significant that its primary upstream signaling pathway involves the PI3k/NF-κB/Akt pathway and is connected to the MAPK-pathway, the apoptotic signaling pathway of mitochondria and the members of the caspase and Bcl-2 families [117]. Triptolide increased tumor cell death by preventing p21 mediated cell-cycle arrest by inducing the concentration of cells in the S phase, blocking the aggregation of cells in the M/G2 phase and doxorubicin’s stimulation of p21. The dysregulation or ablation of glucocorticoid receptor activation in neoplastic plasma cells is linked to drug-resistant steroid treatments. The phosphorylation of glucocorticoid receptor was up regulated by triptolide, which also improved the growth-inhibitory effects of dexamethasone [118].

### 2.5 Tumor metastasis inhibition by triptolide

The primary factor that contributes to the death of a patient with tumor mass is metastasis. In many model systems, including B16F10-melanoma, TSU-bladder cancerous cells, B16, MGC80-3 gastric cancerous cells and MDA-435 breast cancerous cells, Yang S. et al. examined the anti-cancer effects of triptolide. Their findings demonstrated that triptolide prevented investigational dissemination of B16F10 cells to the spleen and lungs of mice and had anti-tumor efficacy against a wide range of cancers that include both wild type or mutant versions of p53 [22]. It has been proven that the CXCR4/SDF-1 signaling pathway contributes to metastasis of tumor. The CXCR4/SDF-1 linkage is blocked by triptolide, which may have an anti-cancer effect by preventing lymphoma cells from migrating to lymph nodes in-vitro [119]. The infiltration of cancerous cells is tightly correlated with the up regulation of the urokinase-type-plasminogen activator receptor (uPAR). Triptolide may at least slightly exert its impact against invasiveness via regulating uPAR expression in gastric cancerous cells by inhibiting activation of NF-κB [120]. One of the most known aggressive cancers is anaplastic-thyroid carcinoma (ATC), which is distinguished by fast growth, initial invasion, and total resistance to modern treatments. In such matrigel model, triptolide significantly reduces growth, angiogenesis, and invading in the TA-K cells of ATC cell line of human. By preventing the p65 subunit’s connection with p300/CBP (CREB binding protein) in the initial stages and depressing the protein level of p65 in the later phase in the cells, triptolide inhibits the transcriptional activity of NF-κB [121]. Moreover, triptolide is a powerful inhibitor of growth and invasion of cancerous cells of colon in-vitro. Triptolide’s anti-metastatic activity may be attributed to the down regulation of many cytokine-receptors, as well as the suppression of VEGF and COX-2 and the optimistic modulation of cell cycle [32]. Triptolide also slows the growth of colon cancerous cells linked to colitis by suppressing the STAT3/JAK and Rac1 pathway [122].

## 3 Clinical trials of triptolide derived drug candidates

Clinical studies using triptolide along with its precursors, such as minnelide and (5R)-5-hydroxy triptolide (LLDT8), have been conducted to treat autoimmune disorders, leukemia, HIV, solid tumors, and rheumatoid arthritis [12]. With limited success, a variety of phase-I clinical trials have really been carried out in individuals who have psoriasis vulgaris, rheumatoid arthritis, systemic lupus erythematosus (SLE), diabetic nephropathy, leukemia, and HIV to evaluate the efficacy of triptolide [123]. Thus, clinical development of (5R)-5-hydroxy-triptolide (LLDT8) and minnelide, which are pursuing ongoing clinical trials, will be our primary emphasis. In preclinical investigations, the drug minnelide, a disodium phosphonooxy-methyl active metabolite of triptolide with improved aqueous solubility, demonstrated remarkable pharmacological properties in the therapy of leukemia, ovarian cancer, pancreatic cancer, liver cancer, and adenocarcinoma of colon [124]. Minnelide can increase the surviving probability in mice having carboplatin resistant/sensitive cancer of ovary when coupled with carboplatin and paclitaxel [125]. Minnelide seems to have the possibility of evolving into a breakthrough pancreatic cancer treatment because it not only killed the cancerous cells but also disrupted the stromal structure and boosted medication contents in malignancies [20]. In 2013, Minneamrita Therapeutics LLC began a phase-I clinical trial (NCT01927965) with 45 participants who had metastatic gastrointestinal lesions to examine the dose-limiting toxicities and higher dose of minnelide. In an effort to demonstrate the impact of minnelide in total 19 individuals with recurrent cancer of pancreas, a controlled clinical phase-II trial (NCT03117920) had been started in 2017. Participants received daily injections of minnelide at a dose of 0.67 mg/m^2^ every day for 21 days, followed by a 7-day break between cycles, until progression of the disease. In 2020, the clinical trial’s findings were supposed to be made public. In order to assess the efficacy, dose progression, and bioavailability of minnelide capsules administered either individually or in conjunction with paclitaxel specifically bound to albumin in total 66 individuals having metastatic solid tumors, an ongoing phase-I clinical research (NCT03129139) was started in October 2017. A phase-I clinical trial (NCT03760523) by Minneamrita Therapeutics LLC was started in 2019 to evaluate the efficacy & dose progression of minnelide in those individuals who have recurrent or refractory acute-myeloid leukemia. A special triptolide derivative with strong immunosuppression and anti-inflammatory properties is (5R)-5-Hydroxytriptolide (LLDT8) [126]. LLDT8 has a broader therapeutic index, reduced toxicity, and improved solubility in water when compared to triptolide [19]. LLDT8 has demonstrated positive therapeutic results in preclinical investigations for the therapy of inflammatory diseases, cerebral reperfusion/ischemia, cancer, lupus-nephritis, autoimmune encephalo-myelitis, soft tissue rejection, and graft versus host disease [126].

Shanghai Pharmaceuticals Holding Co., Ltd. began a phase-II clinical trial (NCT02202395) of LLDT8 in June 2014 for 120 females diagnosed with rheumatoid arthritis who had a poor response to methotrexate (MTX) therapy. These qualified patients received treatment for 24 weeks with a placebo, reduced, moderate, and higher doses of LLDT8 (placebo = MTX, reduced dose = MTX plus 0.25 mg/m^2^/day LLDT8, moderate dose = MTX plus 0.5 mg/m^2^/day LLDT8 and higher dose = MTX plus 1.0 mg/m^2^/day LLDT8). The ACR20 response rates for the various doses were 20%, 46.70%, 50%, and 73.30% respectively. Upper respiratory tract infections, Leukopenia, reproductive problems and liver damage were all side effects of the medication, however the majority of them vanished once the medication was stopped [127]. 160 HIV participants in China with chronic aberrant immune activation were enrolled in phase-II clinical trial (CTR20191397) in July 2019 to assess the effectiveness & safety of LLDT8. This trial is indeed currently in progress. Triptolide has two significant barriers to drug development: strong systemic toxic effects and poor solubility in water. Considering its mode of action, the systemic toxic effects of this compound can be linked to the suppression of RNAPII regulated transcription, a crucial physiological function needed by each cell type. Many attempts to create new triptolide conjugates through structural changes have been attempted [12]. The above mentioned minnelide and LLDT8 are two of them that have been created primarily to solve the solubility issue, while LLDT8 did also show less adverse effects [128].

## 4 Strategies for targeted delivery of triptolide

### 4.1 Triptolide delivery by a small molecule ligand

Using carriers or metabolizing enzymes that are considered to get stimulated in cancerous cells in comparison to healthy cells, several methods had been devised for targeting of triptolide specifically to cancerous cells.

#### 4.1.1 Sugar conjugate of triptolide

##### 4.1.1.1 Transporters of glucose

In latest years, researchers have come to understand the importance of sugar protein linkages in disease and pharmacotherapy owing to a crucial part that sugar expression plays in cell-molecule, cell-matrix and cell-cell exchanges. According to this theory, among the most effective methods for delivering tailored doses of medication while minimizing toxic effects has been demonstrated to be the conjugation of small molecule therapeutics to glucoses. Because of the Warburg phenomenon, cancerous cells overexpress the GLUTs (GLUT3 and GLUT1) in order to maintain their massive development. One glucose-conjugated-cytotoxic product, glufosfamide, is currently being tested in phase-III clinical trial for malignant adenocarcinoma of pancreas. These overly expressed GLUTs in cancerous cells are now considered as possible targets for such preferential killing of cancerous cells by cytotoxic agents in conjunction with glucose (NCT01954992) [129]. The hydroxyl group at C-14 was used to generate an array of triptolide-glucose conjugates (glutriptolides), each of which showed good solubility in water and were conceived and created by Warburg and colleagues. Every one of these glutriptolide derivatives showed no apparent reduction of the ATPase activity (based on DNA) of TFIIH in-vitro because of the steric effects. So far, several glutriptolide derivatives were able to suppress cell growth and cause the degeneration of RPB1 in-vivo. In an animal model of prostate cancer with metastatic spread, glutriptolide, which has better tolerance than triptolide, substantially increased the animals' survival rate [130]. This suggests that glutriptolide is a favorable pro-drug that can target malignant cells more specifically than normal cells while lowering triptolide’s systemic toxic effects.

##### 4.1.1.2 Megalin-receptor

The renal tubule epithelium expresses the **megalin-receptor**, a possible 2-glucosamine target. Carbamate and glucosamine complex of prednisolone is helpful in alleviating renal ischemia/reperfusion injuries and preferentially attacks the kidneys without noticeably compromising bone strength [131]. Zhang’s team used a similar technique to link carbamate and glucosamine to triptolide’s C-14 hydroxyl group, which impacts kidney tissue specifically and has a beneficial effects on renal ischemia/reperfusion injuries [132]. Even when esterases hydrolyzed the carbamate linkage, the liberated triptolide really displayed a high level of cytotoxic effects. The triptolide-glucosamine combination was developed and manufactured using the efficient O-glucosidal link to lessen the toxic effects [133]. Triptolide and glucosamine combination showed greater stability towards renal ischemia/reperfusion injuries at a comparable dose of triptolide.

#### 4.1.2 Cell penetrating peptides (CPPs)

The group of peptides known as cell penetrating peptides (CPPs) includes molecules of 5–30 amino acids, including poly-arginine and the condensed HIV-1 TAT protein. CPPs can transfer numerous physiologically active substituents throughout cell membranes without associating with particular receptors. In both preclinical and clinical research, poly-arginine and arginine-rich peptides are frequently utilized because they can quite easily cross the cytoplasmic membrane of eukaryotic cells [134]. Wang’s team created and synthesized a triptolide and poly-arginine composite that had decreased skin toxic effects in guinea pigs (in-vivo) and HaCaT cells (in-vitro), allowing them to assess the efficacy of the CPP method for triptolide. The ability of triptolide and poly-arginine composite to rapidly infiltrate the corneum barrier and extend to epidermis/dermis in less than 2-h following transdermal delivery further suggests that this compound may be used as a topical medication for managing several inflammatory as well as autoimmune ailments [135].

#### 4.1.3 NAD (P) H-quinone oxidoreductase 1 (NQO1)

Owing to its up regulation in most cancerous cells, NQO1, a cytosolic-reductase that catalyzes the two electron suppression of quinones among favored metabolites, is an anti-carcinogenic agent [136]. The triptolide/quinone propionic acid compound was developed and established by Peng’s lab, and it may be preferentially significantly attenuated by NQO1 in cancerous cells to freely release triptolide [137]. In-vitro, triptolide/quinone propionic acid combination was more effective than healthy hepatocytes over HepG2 cancerous cells, and it demonstrated good anti-hepatocellular neoplasm efficacy in animals without causing significant renal injury. These findings demonstrate the potential of NQO1 as a therapeutic target for the delivery of extremely lethal medicines to cancerous cells.

### 4.2 Triptolide delivery by a macro-molecule ligand

Drugs that contain macro-molecule ligands are more specialized compared to alternatives because they have a greater affinity for binding for specific receptors than small molecular ligands, such as new protein scaffold, aptamers, whole antibodies and antibody fragments [138].

#### 4.2.1 Antibody drug conjugates (ADCs)

ADCs are frequently employed to supply cytotoxic medications, like calicheamicin, to cancerous cells that are up regulating specialized receptors. 6 ADCs, which include those that deliberately target CD30, CD22, CD33, Trop2, HER2, and CD79b, have thus far received approval for the management of various malignancies [139]. In cancerous cells, such as stem cells associated with cancer in mesothelioma and colon-carcinoma, CD26 is abundantly expressed. Lately, using a hetero-bifunctional linkage, Yamada’s team produced the anti CD26 monoclonal antibody as well as its triptolide complex. It was discovered that this compound, yet not its CD26 negative equivalents, demonstrated a dose dependent inhibition of cell growth in CD26 positive cell lines. In allografted mice, the triptolide compound of the anti CD26 monoclonal antibody displayed remarkable anti-tumorigenic effectiveness without significantly increasing cytotoxicity [140]. The FDA has authorized the use of cetuximab, a monoclonal antibody to the EGFR, to treat neck and head cancers such as colorectal cancer and squamous-cell carcinoma. By the suppression of RPII regulated transcriptional activity and methylation of histone H3 lysine, Raz’s team created a triptolide-cetuximab combination (TPL- Cet) with a mean of 5.50 triptolide units per antibody to specifically decrease EGFR overexpression in carcinoma cell lines. TPL-Cet treatment showed greater therapeutic properties and less toxicities I n-vivo when compared to cetuximab and triptolide single therapy or their conjunction [141].

#### 4.2.2 Aptamers

Single-stranded oligonucleotides called aptamers have particular 3-dimensional structures and have a high inclination for binding to their substrates [142]. Aptamers could also supply extremely toxic medications via particular receptors produced on cancerous cells, much as the monoclonal antibodies employed in ADCs. With great affinity, the As1411 (26-mer DNA-single stranded aptamer), which has a G-quadruple design, may preferentially bind nucleolin. Nucleolin is expressed exclusively both intra-cellularly as well as on the cell membrane in many cancerous cells [143]. Two distinct linkers, urethane and succinate, were used to combine triptolide to the AS1411 aptamer to produce certain moieties. Both moieties hence provided an additional triptolide directed delivery method comparable to ADCs by specifically inhibiting the development and proliferation of nucleolin up regulating cancerous cells in-vitro and in-vivo with reduced toxicity and fewer adverse effects [144].

### 4.3 Triptolide loaded nanoparticles based targeted delivery

The precise distribution of medications to targeted tissues or cells has been substantially assisted by nanoscience-created nanosize vehicle based systems for drug delivery. It has many benefits as a significant development in the field of targeted delivery, including improving the ability to specifically target tissues or cells, mitigating chemo-resistance through intracellular transport, and obtaining prolonged and regulated release [145,146]. Moreover, nanoparticles may alter the pharmacokinetic and safety aspects of parent medications, permit targeted drug concentration in tumor sites, and regulate the release of medications to maintain synergic drug concentrations for improved anti-cancer properties. A single nano-vehicle without specialized ligands is insufficient to transport pharmaceuticals to specific areas, despite the nano-significant vehicle’s ability to enhance drug stability and cellular absorption based on local EPR (enhanced permeability and retention) impact. Lately, highly versatile nanoparticles have now been engineered to respond to the micro-environment of tumor or to be coupled to ligands that can specifically bind with cell-specific receptors (antigen, nucleolin and folate receptor) to become the subsequent generation of nano-carrier drug delivery system [147]. Silk-fibroin (SF) protein has been used in a number of drug delivery systems for targeting drug distribution due to its special characteristics, including manufacturing based on water, controllable biodegradation, stabilization effects, biocompatibility and varied material forms. Triptolide loaded silk-fibroin nanoparticles (TP-SFNPs), that have established biocompatibility features as well as pH sensitive profile of drug release, were successfully created by Ding and colleagues. Due to the extended release along with the enhanced stabilization and cellular internalization of the triptolide after its entrapment in NPs, TP-SFNPs demonstrated significantly greater inhibitory activity of colony formation, the capacity to trigger more apoptotic cell death of cancer cells of the pancreas and improved anti-cancer properties as compared to free triptolide. Hepatocellular-carcinoma (HCC), the most prevalent type of recurrent liver cancer and the second most common cause of cancer related death, possesses the worst prognostic effects due to its inadequate response to both chemotherapeutic drugs and directed therapeutic medicines [148]. It is primarily caused by the tumor tissues' inability to retain therapeutic concentrations of the relevant medications, in addition to their extreme toxicity.

**Fig. 3:**
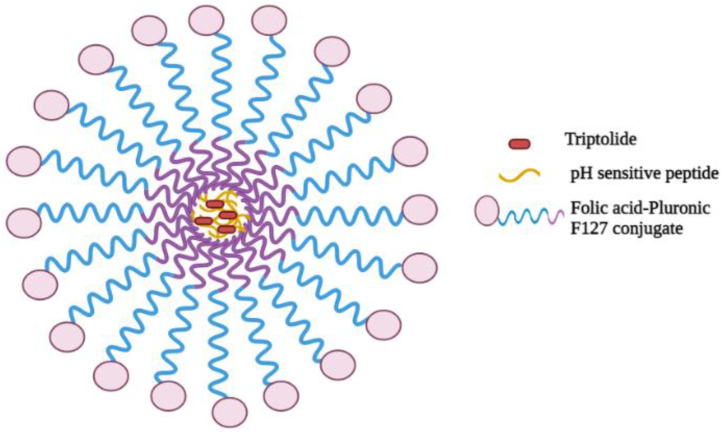
Triptolide loaded pH sensitive nanoparticle designed with folic acid (TP-Nf).

Ling and colleagues fabricated and characterized pH-responsive NPs enclosed with triptolide based folate ligand (TP-Nf). It has particularly dynamic action of triptolide when used against many malicious cancerous microenvironments. Folate based targeted delivery demonstrated improved anticancer effects [149]. The outcome demonstrated that by synthesizing TP-Nf (Fig. 3), triptolide could be administered intravenously and aggregated at tumor site via size dependent nanoparticle aggregation. The pH dependent behavior of peptide is based on TP-Nf which helps it on the on-site release of triptolide (acidic surroundings of HCC-tumor). Moreover, it also improve the cell-specific absorption of triptolide in HCC-tumor for decreased toxicity and greater efficacy. Altogether, the findings imply that functionalized nanoformulations may be useful in lowering the toxic effects of extremely strong medications like triptolide, which could increase treatment effectiveness and, ideally, make it easier to translate these findings into clinical investigations.

One of the main challenges to successful chemotherapy of pancreatic cancerous cells is drug resistance. Even though triptolide signifies greater anticancer activity when compared to gemcitabine and thus can suppress the expansion of chemo resistant pancreatic cancer (CPC) cell lines via a number of different pathways, its clinical interpretation has just been significantly hampered by its non-specific vulnerability to normal tissues. Subsequently, Wang and colleagues created and synthesized a micelle; PEG-PDLLA loaded triptolide with an AS1411-aptamer which can particularly affect chemo resistant pancreatic cancer (CPC) cell lines [150]. They initially attached AS1411-aptamer to HOOC-PEG-PDLLA, and then using the solid dispersion approach, they created micelle (AS1411-PEG-PDLLA) loaded triptolide; AS-PPT. The biophotonic scanning results demonstrated that AS-PPT can concentrate in malignant cells and target CPC in particular [150]. The AS-PPT seems to have more potential anti-cancer effects on cancer cells of the pancreas, according to results of in-vivo anti-cancer activity assessment. That may be due to the unique association between the nucleolin and AS1411-aptamer, which might dramatically increase the cellular absorption of AS-PPT while inhibiting AS-PPT efflux through P-glycoprotein (P-gp). Altogether, this approach offers a fresh, efficient way to treat drug resistant cancerous cells of pancreas.

## 5 Novel triptolide carriers

Triptolide has both immunosuppressive and anti-tumor properties. For the treatment of malignancies, its immunosuppressive activity could be harmful. Methoxy polyethylene-glycol poly (d, l-lactic acid)-block copolymer acts as a carrier in the solvent evaporation process to create a new polymeric micelle system composed of triptolide (TP-PM). The TP-PM was stable over the course of time, exhibited a core-shell shape, and an average size of 78.9 nm. TP-PM injections intravenously have the potential to greatly slow tumorigenesis. Comparative to triptolide, TP-PM seemed to have no impact on the spleen index, thymus index, spleen lymphocyte progression or the serum levels of IL-2 and TNF-α. Although not exhibiting immuno-modulatory effect, triptolide enclosed in polymeric micelles still displays its anti-tumor impact [151]. In one animal model of erythema, Chen J.G. et al. assess the suitability of ethosomes being carriers for the topical therapy of triptolide. Ethanol (45% v/v) and Di-palmitoyl-phosphatidyl choline (2% w/v) were ultrasonically processed for 5 min to create triptolide ethosomes at the favorable condition, resulting in an average particle size of 51.4 nm and 98% encapsulation efficiency. With no penetration time lag, this triptolide ethosomal formulation produced the highest in-vitro 24 h concentration of triptolide, i.e. 83.7%. Compared to other formulations, it more quickly and effectively reduced erythema in-vivo [152].

## 6 Conclusions

Natural substances derived from therapeutic plants or their byproducts have developed into irreplaceable medications like aspirin, paclitaxel, morphine and artemisinin. Triptolide has been thoroughly researched as a therapeutic lead due to its potential pharmacological properties in autoimmune disorders, malignancies and inflammatory conditions, etc. Autophagy and apoptotic processes influence pathological states, normal growth and tissue-homeostasis. These processes of cell death interact with one another in a complicated way. Furthermore, a number of methods exist by which autophagy can promote, inhibit or work with apoptosis-induced signaling pathways. Despite its innate mechanism based general toxicities and poor solubility of moieties of triptolide have impeded its approach towards the clinic, minnelide and LLDT8 have advanced phase-II clinical studies in human. Whereas the poor solubility problem was addressed by minnelide and LLDT8, as well as the toxicities based on systemic mechanism, to a lesser extent, more potent techniques are needed to further lessen the possibility of triptolide side effects and improve its therapeutic efficacy. Targeted administration of triptolide to cancerous or sick cells or tissues has resulted in significant advancements. Glucose-triptolide and antibody-triptolide moieties, two examples of small molecule and macromolecule ligand guided targeted delivery, have been studied and have showed promise for improved efficacy and decreased toxicity in a variety of in-vivo models of cancer. Nevertheless, there has also been some success using nanotechnology to provide targeted administration of triptolide.

## 7 Future prospects

The fate of triptolide along with its compounds as active substances lead to cure a broad range of human ailments, thus appears promising as various targeting mechanisms are to be explored, along with the creation of sustainable resources and useful synthetic approaches for triptolide. Triptolide may soon be more helpful in the management of various cancers, which is what we expect and anticipate.

Cell death mechanisms are interconnected, and their respondents may interact with one another to speed up or slow down cell death [153]. Some novel signaling pathways of cell death, however, may guide the production and research of highly specialized and selective biomolecules for the treatment of cancer that are now emerging. Further studies are needed to show the precise path taken by organic anticancer medications to trigger a particular cell death mechanism and to determine the processes by which a drug shifts from one cell death process into some other. This is particularly pertinent given the importance of autophagy, since this mechanism may promote cell death or viability depending on the many medications employed, combination therapies, and epigenetic as well as genetic modifications in cancerous cells [154]. In hopes of eliminating malignancies, various mechanisms of cell death can be used independently or together as treating cancer in the future. Future studies should concentrate on determining if cancer-stem cells (CSCs) rely on alternate non-apoptotic processes of cell death or on apoptotic ones as we currently understand fewer details regarding cell death processes in CSCs. Knowing this, one may efficiently target CSCs by combining medications that cause a variety of cell death pathways [155]. Moreover, combination therapies may focus on the tumor’s heterogeneous sections, which have distinct epigenetic and genetic markers [156]. It is yet unclear if additional elements, such as the tumor micro-environment [157], changes in metabolic reactions [158] and the immune responses [159], have an impact on the tumor’s heterogeneous cells' response to cell death. Future investigations of cell signaling cascades, biology of systems and genomic techniques will clarify whether distinct chemical markers or mechanisms facilitate or inhibit different types of cell death. This knowledge is essential for effective treatment of cancer.

## Funding

No funding was obtained for this work.

## Ethical approval statement

Being a review article, ethical approval was not required for this study. Moreover, no human beings or animals were used in this study.

## Data availability statement

Being a review article, no data was used for the content described in this article.

## CRediT authorship contribution statement

**Ke Feng:** Investigation, Formal analysis, Data curation, Conceptualization. **Xiaojiang Li:** Writing – review & editing, Writing – original draft, Investigation, Conceptualization. **Yuzhuo Bai:** Writing – original draft, Validation, Software, Conceptualization. **Dawei Zhang:** Visualization, Software, Methodology, Conceptualization. **Lin Tian:** Writing – review & editing, Project administration, Conceptualization.

## Declaration of competing interest

The authors declare that they have no known competing financial interests or personal relationships that could have appeared to influence the work reported in this paper.
